# A Pictorial Dot Probe Task to Assess Food-Related Attentional Bias in Youth With and Without Obesity: Overview of Indices and Evaluation of Their Reliability

**DOI:** 10.3389/fpsyg.2021.644512

**Published:** 2021-03-03

**Authors:** Leentje Vervoort, Maya Braun, Maarten De Schryver, Tiffany Naets, Ernst H. W. Koster, Caroline Braet

**Affiliations:** ^1^Department of Developmental Psychology, Radboud University, Nijmegen, Netherlands; ^2^Department of Developmental, Personality and Social Psychology, Ghent University, Ghent, Belgium; ^3^Department of Experimental Clinical and Health Psychology, Ghent University, Ghent, Belgium; ^4^Faculty of Psychology and Educational Sciences, Faculty Research Support Office, Ghent University, Ghent, Belgium; ^5^Department Health Care (Dietetics), Odisee University College, Ghent, Belgium

**Keywords:** attentional bias, dot probe paradigm, reliability, children and adolescent, obesity

## Abstract

Several versions of the dot probe detection task are frequently used to assess maladaptive attentional processes associated with a broad range of psychopathology and health behavior, including eating behavior and weight. However, there are serious concerns about the reliability of the indices derived from the paradigm as measurement of attentional bias toward or away from salient stimuli. The present paper gives an overview of different attentional bias indices used in psychopathology research and scrutinizes three types of indices (the traditional attentional bias score, the dynamic trial-level base scores, and the probability index) calculated from a pictorial version of the dot probe task to assess food-related attentional biases in children and youngsters with and without obesity. Correlational analyses reveal that dynamic scores (but not the traditional and probability indices) are dependent on general response speed. Reliability estimates are low for the traditional and probability indices. The higher reliability for the dynamic indices is at least partially explained by general response speed. No significant group differences between youth with and without obesity are found, and correlations with weight are also non-significant. Taken together, results cast doubt on the applicability of this specific task for both experimental and individual differences research on food-related attentional biases in youth. However, researchers are encouraged to make and test adaptations to the procedure or computational algorithm in an effort to increase psychometric quality of the task and to report psychometric characteristics of their version of the task for their specific sample.

## Introduction

Different theoretical accounts on problematic eating, overweight, and obesity propose that food stimuli automatically attract visual attention, particularly in individuals with overweight and weight concerns (e.g., Appelhans, 2009; Berridge, 2009; Appelhans et al., 2016; Tanofsky-Kraff et al., 2020). An attentional preference for food is thought to have been evolutionary adaptive in ancient times since it facilitated finding scarce food in the harsh environment, allowing food intake whenever possible, and thus increasing chances for survival. However, in the present obesogenic environment, where energy-dense food is abundantly available, this same mechanism might trigger overeating and increase the risk for overweight and obesity (Robinson and Berridge, 1993, 2008; Blundell and Cooling, 2000; Paquet et al., 2017).

Neurophysiological studies, using brain imaging techniques or eye tracking procedures, indeed support the prediction that individuals with overweight and obesity show stronger attentional biases toward food than individuals with healthy weight, in adults (Hendrikse et al., 2015) and youth (van Meer et al., 2016; Biehl et al., 2020) alike. Evidence stemming from behavioral paradigms, however, is equivocal and shows small to moderate effect sizes at best, especially in youth populations (van Meer et al., 2016; Brand et al., 2020; Hagan et al., 2020; Kemps et al., 2020; Hardman et al., 2021). A possible explanation for these ambiguous results precluding clear conclusions on the role of attentional processes in eating behavior and weight, relates to the considerable methodological inconsistency between studies. Several reaction time tasks are used to measure attentional bias toward food in youth samples, with among others adapted versions of the Stroop task (Stroop, 1935; Braet and Crombez, 2003) and visual search paradigms (Verghese, 2001; Brand et al., 2020). A dot probe detection task (Macleod et al., 1986) with pictures of unhealthy food as targets is the most widely used behavioral paradigm to investigate attentional processes toward food in youth (Kemps et al., 2020).

Several versions of the dot probe procedure have been used to investigate attentional processes in a broad range of problems, like anxiety, depression, addiction, obesity, and problematic eating behavior (Puliafico and Kendall, 2006; Field et al., 2016; Starzomska, 2017; Jiang and Vartanian, 2018; Burris et al., 2019; Rojo-Bofill et al., 2019; Kemps et al., 2020), encompassing a large variety in task characteristics like presentation times, stimulus types (verbal, pictorial), stimulus alignment (vertical, horizontal), instruction (detect probe, categorize probe orientation), and number of trials. Despite this procedural variability, the basic set-up of a trial in the dot probe is rather straight-forward. In general, a pair of stimuli is presented simultaneously on the screen. In target trials, one of these stimuli is considered relevant (e.g., a picture of food in obesity research), while the other is neutral (e.g., a picture of household appliances). Presentation duration of the stimulus pair is typically short (e.g., 200–250 ms: Godijn and Theeuwes, 2002; Born et al., 2011) in an attempt to trigger and capture fast, automatic or uncontrolled processes. In research with youth samples, presentation duration is generally 500 ms (Shechner et al., 2012; Dudeney et al., 2015). Immediately after the stimulus pair is removed from the screen, a probe (e.g., a white dot) is presented at one of the two stimulus locations. If this probe appears at the location of the relevant target stimulus, the trial is considered “congruent.” If the probe appears at the opposite location of the target stimulus, the trial is considered “incongruent.” Participants are asked to indicate as fast as possible in which location the probe appears using one of two response keys. Irrespective of the procedural variability, all studies start from the same theoretical assumptions that participants will consistently react faster to a stimulus that appears in a location where their attention was already focused on than to a stimulus appearing in an unattended location (Posner, 1980). This assumption has given rise to the calculation of an attentional bias score (ABS) as the mean difference score between reaction times on incongruent and congruent trials (RT incongruent–RT congruent, e.g., Macleod et al., 1986). Applied to attentional biases in eating behavior and weight problems, an attentional bias toward food would therefore be visible in positive attentional bias scores, while attentional avoidance of food would be visible in negative attentional bias scores. This “traditional” ABS is still the most frequently used behavioral index of biased attention. However, the index shows considerably problematic psychometric characteristics in terms of reliability and validity (Schmukle, 2005; Ataya et al., 2012a,b; Field and Christiansen, 2012; Rodebaugh et al., 2016; Chapman et al., 2019; McNally, 2019).

While the dot probe paradigm is frequently used tool in experimental research allowing to test group differences, its adequacy to assess individual differences in correlational clinical research has been questioned repeatedly. One major objection against its use is driven by its unacceptably low levels of reliability (Schmukle, 2005; Ataya et al., 2012a,b; De Schryver, 2018; Parsons et al., 2018; Chapman et al., 2019; MacLeod et al., 2019; Van Bockstaele et al., 2020). This observation is related to the so-called Reliability Paradox (De Schryver et al., 2016; Hedge et al., 2018; Goodhew and Edwards, 2019). Experimental research seeks to minimize differences between individuals in experimental conditions aiming to maximize between-group differences following experimental manipulation. A reliable task in experimental research is a task with low measurement error that yields the most homogeneous performance in one group compared to the homogeneous performance in the other group. Between-group differences can then be attributed to the experimental manipulation rather than to individual differences. In contrast, correlational research seeks to maximize interindividual differences in heterogeneous samples. A reliable task in correlational research depends on the extent to which the instrument consistently ranks individuals based on the variance in their true-score variance (Cronbach, 1957). These diverging takes on reliability in experimental vs. correlational research, based on the different aims of both research domains, has its implications on the transfer of experimental paradigms to correlational studies. Reliabilities in correlational research generally do not reach the cut-off guidelines used in experimental research, let alone the 0.90 that is recommended for making inferences about individuals (Rodebaugh et al., 2016). However, if low reliability is mainly due to a lack of true-score variance rather than a large amount of error-score variance, a (correct) use of the instrument might not be problematic (De Schryver et al., 2016, 2018a).

Generally, in individual differences research, reliability, and validity of reaction time-based indices of attentional bias, including the ABS calculated from the dot probe, are not routinely reported (Green et al., 2016; Rodebaugh et al., 2016; Parsons et al., 2018; Goodhew and Edwards, 2019). This practice stands in stark contrast with the strict psychometric requirements posed to variables based on questionnaire scores (Vasey et al., 2003; Parsons et al., 2018) and discords with the prerequisite of reliable instruments for effective research (Lebel and Paunonen, 2011; De Schryver et al., 2016, 2018a). The scarce psychometric reports of tasks based on the dot probe procedure that have been published, have repeatedly shown unacceptable low levels of reliability of the traditional ABS, both in adult populations (Schmukle, 2005; Lebel and Paunonen, 2011; Rodebaugh et al., 2016; Parsons et al., 2018; Chapman et al., 2019; Hagan et al., 2020; Molloy and Anderson, 2020) and in youth (Britton et al., 2013; Brown et al., 2014; Waechter et al., 2014; Fu and Perez-Edgar, 2019). Furthermore, instead of reporting reliability of the ABS, several reliability reports (e.g., Vervoort et al., 2011; Haft et al., 2019) are often limited to the reliability of the unprocessed reaction times (RT) which are rather stable and consistent among participants. However, general reaction time, thus being (relatively) fast or slow, does not provide information of attentional bias: to infer about attentional bias, comparison between responses on congruent vs. incongruent trials is needed. Therefore, since reliability indices should be referring to the outcome of interest (i.e., ABS as index of attentional bias, not general RT as index of processing speed), this practice is non-committal (Kruijt et al., 2016; Parsons et al., 2018).

Aiming to improve psychometric properties of the dot probe task, researchers have been considering adaptations in the task design and the computation of the attentional bias index (Price et al., 2015). Procedural adaptations include using idiosyncratic, personally relevant rather than general stimuli (Christiansen et al., 2015; van Ens et al., 2019), or prolonging stimulus presentation up to 3000–5000 ms (Waechter et al., 2014; van Ens et al., 2019). However, none of these procedural adaptations managed to establish adequate reliability (Jones et al., 2018). In addition to such procedural adaptations, several scholars examined different computational methods to calculate alternative indices of biased attention, exploring their impact on validity and reliability (Price et al., 2019). Reliability might be improved when traditional ABS are calculated by using only bottom-target trials (instead of both top- and bottom-target trials) in vertically oriented dot probe tasks (Price et al., 2015; Aday and Carlson, 2019), although this approach is not always successful (Jones et al., 2018). Simply distinguishing between vigilance (difference between congruent relevant trials and neutral trials) and disengagement (difference between incongruent relevant trials and neutral trials) aspects of attention also failed to improve reliability (Koster et al., 2004; Waechter et al., 2014). However, adopting a response-based approach to vigilance and disengagement might result in higher reliability scores (Evans and Britton, 2018).

Alternatively, researchers challenged the assumption of attentional bias as a stable concept underlying the traditional calculation of the ABS, and suggest that attentional bias is a dynamic process fluctuating over time, with attention being switched back and forth between relevant and neutral stimuli (Iacoviello et al., 2014; Zvielli et al., 2014, 2015; Rodebaugh et al., 2016; McNally, 2019; Hardman et al., 2021). To account for this dynamic in attention, Iacoviello and colleagues proposed the attention-bias variability score (ABVS, Iacoviello et al., 2014). The ABVS is computed by grouping the dot-probe trials in sequential bins, calculating the ABS for each bin, and dividing the SD of ABS across all bins by the mean RT of the total task. The resulting ABVS is an index of stability of attention biases, with increasing ABVS thus suggesting more fluctuation in attentional biases toward and away from relevant stimuli over time. The ABVS, however, does not allow to differentiate between the approach and avoidance aspects of these dynamics, which might be of particular interest in eating behavior (Liu et al., 2019a,b; Hardman et al., 2021). Zvielli et al. (2015) proposed the trial-based bias-score (TL-BS) as a way of simultaneously distinguishing direction of attention and dynamic variability over time. The TL-BS is computed by forming pairs of congruent and incongruent trials on the basis of temporal proximity and subtracting the RT of the congruent trial from RT of the incongruent trial for each pair. From the resulting time-series of TL-BS's, five indices of biased attention can then be derived for each participant: The mean and peak of all the positive TL-BS's in the series (TL-BSpos), the mean and peak of all negative TL-BS's in the series (TL-BSpos), and a TL-BS variability index, computed as the mean absolute distance across the whole series of TL-BS's. Applied to attentional biases in eating behavior and weight problems, mean TL-BSpos is considered to reflect the amount of approach bias toward food, mean TL-BSneg the amount of avoidance bias away from food, peak TL-BSpos and peak TL-BSneg, the maximum expression of bias toward vs. away from food, respectively and TL-BS variability the amount of fluctuation between bias toward and away from food over time (Liu et al., 2019a,b). ABV and TL-BS scores are thought to show a cyclic pattern, reflecting one's attention switching toward and away from the relevant stimuli over time (Iacoviello et al., 2014; Zvielli et al., 2015). In adult samples, reliability of these dynamic indices of attentional bias is superior compared to traditional indices (Zvielli et al., 2015; Rodebaugh et al., 2016; Molloy and Anderson, 2020). On top of the general theoretical assumption underlying the traditional approach to the dot probe (faster RT to a stimulus appearing in the already attended location, i.e., faster RT in congruent than in incongruent trials), the dynamic approach adds the assumption that the RT differences between congruent and incongruent trials may vary meaningfully over time. Higher variability over time is thought to reflect pathological attention switching while a more stable pattern of attention orienting is thought to be adaptive (Zvielli et al., 2015). However, when accounting for general variability in RT, these assumptions may not hold (Zvielli et al., 2015; Kruijt et al., 2016; Carlson and Fang, 2020). In a monte-carlo simulation it was shown that the dynamic indices are likely to capture not only information of attentional bias, but also of measurement error (Kruijt et al., 2016; McNally, 2019). Furthermore, when accounting for general variability in RT in adult samples, the superior reliability of the dynamic indices is also lost (Carlson and Fang, 2020).

Research on reaction-time paradigms has illustrated that RT-based indices are largely influenced by general response speed (Fazio, 1990; Faust et al., 1999; Greenwald et al., 2003; Glashouwer et al., 2013; De Schryver et al., 2018b), with larger indices (independent from direction) for individuals with slower reaction times across the task. General response speed and RT-variability are found to decrease from childhood over adolescence to adulthood, while increasing from then on (Dykiert et al., 2012; Adleman et al., 2016). This developmental trajectory might typically be associated with even smaller indices in youth samples compared to adult samples. An innovative approach to compute meaningful indices based on RT based data while accounting for differences in general response speed, has been proposed recently by De Schryver and de Neve (2018). They suggested the Probability Index (PI) as an index for the Implicit Associations Test (I.A.T., Greenwald et al., 1998), with enhanced reliability over traditional I.A.T.-indices (De Schryver et al., 2018b). The PI reflects the probability that a randomly chosen response on a congruent trial is faster than a randomly chosen response on an incongruent trial. Although not earlier used to index attentional processes, this approach can easily be transferred to the dot probe paradigm, with higher PI's reflecting stronger attentional bias toward the relevant stimuli. Applied to attentional biases in eating behavior and weight problems, an attentional bias toward food would therefore be visible in higher PI's (PI > 0.05), while attentional avoidance of food would be visible in lower PI's (PI < 0.05).

Irrespective of the decennia-old abundance of literature discussing the limitations of behavioral reaction time paradigms to assess individual differences in biased attention and the static or dynamic nature of attentional processes (Schmukle, 2005; Field et al., 2016; Rodebaugh et al., 2016; Goodhew and Edwards, 2019), scholars investigating attention bias to food and developers of innovative theory-based interventions targeting these processes (Eichen et al., 2017; Kemps et al., 2020) nevertheless keep on using reaction time tasks, including the dot probe task, in their work, often without evaluating psychometric properties of the specific test in the specific study sample. This practice urged the effort to establish evidence for the use of a pictorial dot probe task to investigate food-related attentional biases in youth with and without obesity. The present study will take on this challenge, by scrutinizing psychometric properties of traditional as well as innovative indices of the dot probe: the traditional ABS, the dynamic TL-BS, and the probabilistic PI. It will be examined whether food-related attentional biases can be meaningfully and reliably assessed using the different bias indices computed from responses on a pictorial dot probe, in a sample of youth with and without obesity. The applicability of this specific task procedure will be evaluated for experimental research, by testing group differences, and for individual differences research, by calculating correlations of the indices with weight (Greenwald et al., 2003). Reliability (in terms of performance stability) of the indices will be evaluated by comparing performance in the first part of the task with performance in the second part.

## Methods

### Sample

Participants of the present study were 337 children and adolescents (65% girls), aged between 7 and 19 (*M* = 14, *SD* = 2.59). 59.64% of the participants were recruited in the WELCOME-project (ISRCTN14722584, Naets et al., 2018), a RCT evaluating executive functions training for weight control in youth. Children and adolescents (age *M* = 14, *SD* = 2.45) in this subsample were all obese (adjusted BMI: *M* = 183.47, *SD* = 35.17). The remaining participants (age *M* = 13, *SD* = 3.36) were recruited in convenience samples by Master students at Ghent University under supervision of LV and TN. They were all normal weight (adjusted BMI: *M* = 99.18, *SD* = 7.95). This sample size was justified by data availability: all data that were collected at Ghent University, using this particular dot probe procedure between 2017 and 2020 were used. As such, the sample size is sufficiently large to detect group differences of *d* = 0.4, which is considered the smallest effect size of interest in psychology (Lakens et al., 2018), and reach 80% power for alpha = 0.05 (Brysbaert, 2019). Both data collection procedures were approved by the IRB (UZGent 2017/0305 and UGent FPPW 2019/79).

### Weight

To index weight status in a developmentally appropriate way, age and sex adjusted Body Mass Index (adjBMI) was calculated by dividing measured BMI (weight in kg/squared length in cm) by norm BMI for age and sex, and multiplying this by 100. Norm BMI for age and sex was determined as the 50th percentiles of the BMI for age and sex based normative data. An adjBMI equal to or smaller than 85% is considered underweight, equal to or >120% as overweight, equal to or >140% as moderate obesity, equal to or >160% as extreme obesity.

### Dot Probe Task

Attentional bias toward food-related stimuli was measured using a pictorial version of the dot probe task (Macleod et al., 1986) with food and neutral stimuli selected from the Food-pics database (www.food-pics.sbg.ac.at Blechert et al., 2014). Picture pairs were matched for visual complexity, brightness, and contrast. The data were collected by means of a dedicated JavaScript web application that runs in the browser. Stimulus presentation routines were handled by a custom Python-based backend. The software was developed by ImplicitMeasures.com, a spin-off company of Ghent University (Belgium). After presentation of a white fixation cross in the middle of the screen, a picture pair is presented for 500 ms, one to the left and one to the right of the center. This procedure was chosen to match earlier work on food-related attentional bias in adult samples (Kemps et al., 2014). Immediately following the pictures, a white dot appears on one of the locations (either left or right). Participants are asked to react to the dot by pressing “e” on a keyboard when the dot appears on the left side, and pressing “i” when it appears on the right side. In total, 140 trials are presented, of which 10 neutral-neutral trials as practice trials, 16 food-neutral pairs each presented four times, resulting in 64 experimental trials. The remaining trials are filler trials presenting two neutral pictures (neutral-neutral trials) (Naets et al., 2018).

#### Indices for Attentional Bias

Trials with RT outliers (trial RT < 200 ms or > 1.500 ms) or incorrect responses were excluded.

*Traditional Attentional Bias Score (ABS)* (Macleod et al., 1986) was calculated by subtracting RT of congruent trials from RT of incongruent trials, such that ABS>0 are indicative of bias toward food and ABS<0 of bias away from food. Additionally, the absolute value of ABS is taken, so higher values indicate stronger effects, either toward or away from food.

*Dynamic indices* (Zvielli et al., 2015) were conceptualized as TL-BS parameters. The TL-BS is computed by subtracting the RT of a congruent trial from RT of its incongruent counterpart for pairs of trials that were in close temporal proximity (not further than five trials apart). Mean and peak values of all the positive TL-BS's in the series are indicative of bias toward food. Mean and peak values of all the negative TL-BS's are indicative of bias away from food. The TL-BS variability value indexes the amount of fluctuation between bias toward and away from food over time.

To accommodate for the expected response speed artifact, the *Probability Index* (PI) (De Schryver and de Neve, 2018) is calculated using the following formula so that higher PI is indicative of attentional bias toward food:

$$\begin{array}{l} PI=\;\frac{U}{\left(N_{CT}\times N_{IT}\right)}\;, \end{array}$$

with *U* being the Wilcoxon test statistic for two samples. To ignore direction of the effect, the absolute value of PI, abs(PI-0.5), is taken. Again, higher values indicate stronger effects, either toward or away from food.

### Criteria for Evaluating the Indices and Analytic Plan

Evaluating the different indices was done stepwise, vis-à-vis the considerations below.

#### Independence of General Response Speed

RT-based effects, as the ABS and the TL-BS are known to be inflated for individuals responding slowly (Fazio, 1990; Faust et al., 1999; Greenwald et al., 2003; Glashouwer et al., 2013; De Schryver et al., 2018b). Since general response speed gets faster from childhood to adolescence (Dykiert et al., 2012; Adleman et al., 2016), RT-based effects are expected to be negatively correlated with age. To maximize the independency of the different indices and the measure for general response speed, the average RT of the neutral trials as a measure of general response speed was chosen. A positive correlation between these indices and general response speed on neutral trials can therefore be expected. Such a correlation is expected to be non-significant when using the PI. It would be preferable for an index of attentional bias to minimize the correlation with general response speed and age.

#### Reliability of the Indices

Split-half reliability of these indices (ABS, TL-BS, and PI) will be estimated by Pearson correlations between index scores calculated in both test halves, for the total group and for both weight status groups separately. To test if reliability was influenced by general response speed, linear models predicting performance in first test half by reliability in second test half and RT were computed.

#### The Dot Probe in an Experimental Context

Based on the theoretical assumptions on problematic eating and overweight and obesity, a significant difference can be expected between youth with obesity and youth with normal weight on their reaction to food vs. neutral stimuli. This will be tested using linear mixed models (LMM, Field, 2012) with RT as dependent variable, fixed factors weight status, trial type (both effect coded), and the interaction term weight status x trial type and with participant as random factor (Model 1). In addition to raw RT, logRT will also be tested to account for the typical skewness of the raw RT distribution (Model 2). If the pictorial dot probe would be suitable for use in experimental research in this youth sample, a significant interaction effect between trialtype and weight status would emerge.

In order to evaluate whether the attentional biases indices are capable of predicting weight status, nine separate Linear Probabilistic Models (LPMs) with weight status group as dependent variable (dummy coded), and the indices as independent variables will be reported. To control for general response speed, mean RT on neutral-neutral trials will also be added as between-subject variable. If an index would be a meaningful measurement of food-related attentional bias in experimental research for this youth sample, weight status would be significantly predicted by the index, with no significant effect of general response speed.

#### The Dot Probe Indices in An Individual Differences Context

Because attentional bias for food is thought to be stronger in individuals with higher weight, the linear association between the attention bias indices and adjusted BMI will be estimated by Pearson correlations. If an index would be a meaningful individual differences variable, significant positive correlations would emerge.

## Results

### Descriptives

Table 1 shows the descriptives of the seven attention bias indices, for the total sample (*n* = 337), and both weight groups separately (obesity: *n* = 201, normal weight, *n* = 136).

**Table 1:** Descriptives of the different attention bias indices.

	**Total sample**		**Obesity**		**Normal Weight**	
	**M**	**(SD)**	**M**	**(SD)**	**M**	**(SD)**
ABS	0.96	(35.48)	0.08	(36.78)	2.24	(33.59)
mean TL-BSpos	120.01	(58.98)	122.13	(60.50)	116.94	(56.80)
peak TL-Bspos	428.73	(232.34)	423.49	(229.50)	436.35	(237.06)
mean TL-Bsneg	−119.07	(55.13)	−124.08	(55.40)	−111.81	(54.11)
peak TL-Bsneg	−442.88	(229.87)	−461.05	(227.11)	−416.50	(232.13)
TL-BS variability	169.86	(72.46)	173.22	(71.99)	164.97	(73.13)
PI	0.50	(0.07)	0.51	(0.08)	0.50	(0.07)

### The Dot Probe in an Experimental Context: Group Differences Between Obesity and Normal Weight

Table 2 shows the results of the LMMs. The main effects of congruency and weight status were not significant when predicting raw RT. Also, the crucial interaction term between those two factors was not significant. The same observation was made when predicting log RT. In other words, there is no evidence that RT depends on the congruency of the trials, not even for the obese weight group.

**Table 2:** Regression coefficients of the Linear mixed models.

	**Model 1: raw RT**			**Model 2: logRT**		
Fixed effects1^*^						
	**Estimate**	**Std. error**	***t***-value1^**^	**Estimate**	**Std. error**	***t*****-value**
(Intercept)	522.44	5.90	88.62	6.21	0.01	612.06
Trial type	0.71	0.98	0.72	0.00	0.00	1.02
Weight status group	−6.59	5.90	−1.12	−0.01	0.01	−0.94
Trial type × weight status group	0.34	0.98	0.35	0.00	0.00	−0.21

* *Trial type and Weight status group are effect coded*.

** *No p-values are reported by the lmer-package: absolute t-values > 2 are considered significant for alpha = 0.05*.

### Independence of Mean Response Speed and Age

Table 3 shows Pearson correlations of the attention bias indices with age and mean reaction time on neutral-neutral trials. Traditional ABS, PI and abs(PI-0.5) were not significantly related to mean RT; absolute ABS and TL-BS indices, however, were, with correlations indicating medium effect sizes for absolute value ABS and peak TL-BS, and large effect sizes for mean TL-BS and TL-BS variability. PI and abs(PI-0.5) were not significantly related to age. The other indices, however, were, with correlations indicating small to medium effect sizes.

**Table 3:** Correlation of attention bias indices with age and mean neutral-neutral RT.

	**Age**	**RT (neutral-neutral)**
ABS	0.171^*^	−0.09
Absolute value ABS	−0.201^**^	0.311^**^
mean TL-BSpos	−0.321^**^	0.651^**^
peak TL-BSpos	−0.221^**^	0.461^**^
mean TL-BSneg	0.391^**^	−0.681^**^
peak TL-BSneg	0.221^**^	−0.411^**^
TL-BS variability	−0.351^**^	0.651^**^
PI	0.12	−0.08
Abs(PI-0.5)	0.08	−0.01

* *p < 0.05*;

** *p < 0.01*.

### Reliability

Table 4 shows the split-half reliability estimates for the nine attention bias indices, for the total sample, and the weight status groups separately. Split-half reliability was only significant for mean and peak TL-BSpos, mean TL-BSneg, and TL-BS variability. Table 5 shows, however, that when the association between the scores on both test-halves is controlled for general response speed (on neutral trials), no significant associations between the two halves remain.

**Table 4:** Split-half correlation as index of reliability for the different attentional bias indices.

	**Total sample**	**Obesity**	**Normal weight**
ABS	0.05	0.03	0.09
Absolute value ABS	−0.02	0.01	−0.07
Mean TL-Bspos	0.291^**^	0.261^*^	0.341^**^
Peak TL-Bspos	0.241^**^	0.20	0.321^*^
Mean TL-Bsneg	0.281^**^	0.291^*^	0.30
Peak TL-Bsneg	0.13	0.10	0.19
TL-BS variability	0.431^**^	0.391^**^	0.451^**^
PI	0.08	0.02	0.16
Abs(PI-0.5)	0.01	−0.02	0.07

* *p < 0.05*;

** *p < 0.01*.

**Table 5:** Linear models predicting performance in the first task half by performance in the second task half and general response speed.

	**mean TL-BSpos**		**Peak TL-BSpos**		**Mean TL-BSneg**		**TL-BS variability**	
	**Std coeff**	**0.01 CI**	**Std coeff**	**0.01 CI**	**Std coeff**	**0.01 CI**	**Std coeff**	**0.01 CI**
(Intercept)	0.00	[−0.15, 0.15]	0.00	[−0.16, 0.16]	0.00	[−0.16, 0.16]	0.00	[−0.14, 0.14]
Performance 2nd half	0.05	[−0.12, 0.22]	0.09	[−0.08, 0.27]	0.08	[−0.11, 0.26]	0.20	[0.02, 0.37]
RT (neutral-neutral)	0.50	[0.33, 0.67]	0.36	[0.19, 0.54]	−0.38	[−0.56, −0.20]	0.41	[0.24, 0.59]

### The Dot Probe in an Experimental Context: Predicting Weight Status

Table 6 shows the linear models predicting weight status. Although mean TL-BSneg was found to be a significant predictor of weight status (*B* = −0.001, *SE* = 0.001, *t*(328) = −2.03, *p* = 0.04) none of the models predicting weight status by attention and general response speed reached significance (all *F*s <1, all *p*s > 0.05), with multiple R-squared and adjusted R-squared ranging between *R*^2^ = 0.002 (for mean TL-BSpos) and *R*^2^ = 0.01 [for mean TL-BSneg and Abs(PI-0.5)], and between adj*R*^2^ = −0.004 (for mean TL-BSpos) and adj*R*^2^ = 0.008 [for mean TL-BSneg and Abs(PI-0.5)], respectively.

**Table 6:** Linear models predicting weight status.

	**Index of attentional bias**							
	**ABS**				**Absolute value ABS**			
	**Estimate**	**St. error**	***t*-value**	***p***	**Estimate**	**St. error**	***t*-value**	***p***
(intercept)	0.47	0.14	3.40	<0.001	0.48	0.14	3.44	<0.001
Index	0.00	0.00	−0.46	0.64	0.00	0.00	1.07	0.29
RT on neutral trials	0.00	0.00	0.90	0.37	0.00	0.00	0.57	0.57
	*R*^2^ 0.003		adj*R*^2^ −0.003		*R*^2^ 0.006		adj*R*^2^ −0.0001	
	*F*(2, 331) = 0.55	*P* = 0.58			*F*(2, 331) = 1.02	*P* = 0.36		
	**Mean TL-BSpos**				**Peak TL-BSpos**			
	**Estimate**	**St. error**	*****t***-value**	*****p*****	**Estimate**	**St. error**	*****t***-value**	*****P*****
(intercept)	0.51	0.16	3.25	0.001	0.47	0.15	3.16	0.002
Index	0.00	0.00	0.40	0.69	0.00	0.00	−0.94	0.35
RT on neutral trials	0.00	0.00	0.30	0.76	0.00	0.00	1.09	0.28
	*R*^2^ 0.002		adj*R*^2^ −0.004		*R*^2^ 0.004		adj*R*^2^ −0.002	
	*F*(2, 328) = 0.35	*p* = 0.70			*F*(2, 328) = 0.71	*p* = 0.49		
	**Mean TL-BSneg**				**Peak TL-BSneg**			
	**Estimate**	**St. error**	*****t***-value**	*****p*****	**Estimate**	**St. error**	*****t***-value**	*****p*****
(intercept)	0.59	0.16	3.81	<0.001	0.50	0.15	3.38	<0.001
Index	−0.001	0.00	−2.03	0.04	0.00	0.00	−1.57	0.12
RT on neutral trials	0.00	0.00	−0.83	0.41	0.00	0.00	0.04	0.97
	*R*^2^ 0.01		adj*R*2 0.008		*R*^2^ 0.009		adj*R*^2^ 0.003	
	*F*(2, 328) = 2.33	*p* = 0.09			*F*(2, 328) = 1.51	*p* = 0.22		
	**TL-BS variability**							
	**Estimate**	**St. error**	*****t***-value**	*****p*****				
(intercept)	0.51	0.15	3.37	<0.001				
Index	0.00	0.00	0.70	0.48				
RT on neutral trials	0.00	0.00	0.11	0.91				
	*R*^2^ 0.003		adj*R*^2^ −0.003					
	*F*(2, 328) = 0.52	*p* = 0.59						
	**PI**				**Abs(PI-0.5)**			
	**Estimate**	**St. error**	*****t***-value**	*****p*****	**Estimate**	**St. error**	*****t***-value**	*****p*****
(intercept)	0.33	0.24	1.40	0.16	0.39	0.14	2.77	<0.01
Index	0.25	0.36	0.68	0.50	1.17	0.60	1.96	0.05
RT on neutral trials	0.00	0.00	1.00	0.32	0.00	0.00	0.96	0.34
	*R*^2^ 0.004		adj*R*^2^ −0.002		*R*^2^ 0.01		adj*R*^2^ 0.008	
	*F*(2, 331) = 0.67	*p* = 0.51			*F*(2, 331) = 2.37	*p* = 0.10		

### The Dot Probe in an Individual Differences Context: Associations Adjusted BMI

Table 7 shows Pearson correlations of the attention bias indices with adjusted BMI. None of the correlations reached significance.

**Table 7:** Correlation of attention bias indices with adjusted BMI.

	**Adjusted BMI**
ABS	0.03
Absolute value ABS	0.02
mean TL-BSpos	−0.03
peak TL-BSpos	−0.04
mean TL-BSneg	−0.03
peak TL-BSneg	−0.05
TL-BS variability	0.01
PI	0.06
Abs(PI-0.5)	0.05

## Discussion

The present study investigated whether attentional bias toward food could be meaningfully assessed in a youth sample of children with and without obesity, using a pictorial version of the dot probe task. The rationale for this study, was grounded in the widespread practice to use the dot probe procedure to measure and modify food-related attentional bias, both in experimental laboratory studies and in clinical intervention studies (Kemps et al., 2020), despite ample reports of debatable psychometric properties of different dot probe tasks (Schmukle, 2005; Ataya et al., 2012a,b; Parsons et al., 2018; Chapman et al., 2019; MacLeod et al., 2019). Attempting to save the case for the dot probe, we sought to examine the psychometric properties of the task in a comprehensive manner by scrutinizing different indices of attentional bias that could be calculated from our version of the task, with pictures selected from the Food-Pics database (Blechert et al., 2014) administered to children and adolescents aged 7–19, with and without obesity (for a complete description of the task, see Naets et al., 2018). We evaluated the indices meticulously and thoroughly, by testing whether they would be independent of general response speed, whether they would lead to reliable scores in the present sample, whether they could differentiate between different groups for whom we expected differential performance based on theory (i.e., normal weight vs. obesity), and whether they would meaningfully be associated with individual differences in weight. We will discuss the findings on each of these domains.

Because it is known that RT-based scores are often inflated in individuals who are slower in responding (Fazio, 1990; Faust et al., 1999; Greenwald et al., 2003; Glashouwer et al., 2013; De Schryver et al., 2018b), and response speed increases from childhood to adolescence (Dykiert et al., 2012; Adleman et al., 2016), it is imperative to estimate the association of the attentional bias index with general response speed and age. The probabilistic indices aim to account for differences in general response speed, and in this study, they achieved this aim: PI and abs(PI-0.5) were not significantly correlated with mean RT on neutral trials, nor with age. The traditional ABS showed no significant correlation with RT either, but correlated significantly with age: traditional ABS scores were higher for older than for younger participants. However, both abs(ABS) and all dynamic indices, were strongly correlated with both RT and age, with correlations indicative of medium to large effect sizes for RT and small to medium effect sizes for age. The linear models predicting TL-BS in the first test half by mean RT and performance in the second test half, supported the conclusion that TL-BS indices are significantly determined by response speed. The criterium of independence from general response speed was only met by the traditional ABS and the PI scores. Only the PI scores showed independence from age.

Reliability was estimated by comparing the indices calculated on the first half of the task with the indices calculated on the other half. Both traditional and probabilistic indices showed near-zero correlations. The dynamic indices (except the peak TL-BSneg) showed higher and significant correlations, comparable to those reported by their developers (Zvielli et al., 2016). However, the estimates of reliability still did not reach conventional cut-off guidelines (Cronbach, 1951), let alone the recommended 0.90 for individual design research (Rodebaugh et al., 2016). Furthermore, since the linear models predicted that performance in the first test half was largely and significantly determined by reaction time, it can be concluded that these inflated correlations reflect stability in general response speed rather than stability in the attentional process. The criterium of acceptable reliability was met by none of the indices.

Based on theoretical assumptions that food-related attentional processes differ between people with and without eating and weight problems (Appelhans, 2009; Berridge, 2009; Appelhans et al., 2016; Tanofsky-Kraff et al., 2020), significant between-group differences would need to emerge on meaningful indices of attentional bias. However, in the LMMs, there was no support for differential performance on the dot probe task between youth with and without obesity in the present study, irrespective of the index. Furthermore, in the linear models predicting weight status, only mean TL-BSneg emerged as a significant predictor. However, the model in question (as the other models), did not reach significance, with no more than 1% of variance explained. The criterium of differential performance between groups or predictive validity in terms of group membership was met by none of the indices. As such, there was no evidence that the dot probe task as administered in the present study, could meaningfully be used to assess group differences in experimental research with youth with and without obesity.

Theory (Appelhans, 2009; Berridge, 2009; Appelhans et al., 2016; Tanofsky-Kraff et al., 2020) also states that food-related attentional biases would get stronger in individuals with increasing weight. Although there is some debate on whether this attentional bias would reflect increased approach or increased avoidance (Liu et al., 2019a,b; Hardman et al., 2021), effects are predicted to be significantly correlated with weight parameters. However, none of the indices correlated significantly with adjusted BMI. As such, there was no evidence that the dot probe task as administered in the present study, could meaningfully be used to assess individual differences in food-related attentional bias.

The sobering results of the present study cast doubt on the use of the dot probe procedure as an instrument for assessing maladaptive attentional processes in problematic behavior or psychopathology. However, this need not be the deathblow of the dot probe procedure, since several issues need to be taken into account. Here, the results only pertain to this specific version of the test, with these specific procedural characteristics (e.g., stimuli, presentation times, …), When using this test set up, administered to this specific sample (children and adolescents with and without obesity, aged 7–19), to compute these specific indices (ABS, TL-BS, PI), we were unable to provide evidence for the task's applicability to assess food-related attentional biases. However, these conclusions pertain only to this test version, in this sample in this context (De Schryver et al., 2018a), and preclude generalization to other versions of the task in other samples and contexts. Adaptations to the task, that might be worth trying, could be, among others, the use of personally relevant stimuli (Christiansen et al., 2015) or prolonging presentation time (Waechter et al., 2014). Although these adaptations did not result in increased reliability in adult samples (Jones et al., 2018), they were not evaluated in younger samples. Given the impact of test length on reliability (Gulliksen, 1950; Morera and Stokes, 2016; McNally, 2019), one might consider administering more trials. However, the boredom which might be triggered by long repetitive tasks, could potentially be detrimental to attention (Eastwood et al., 2012; Hunter and Eastwood, 2018), especially in younger samples (Hsu et al., 2020). The optimal number of trials, balancing effects on reliability and boredom, still needs to be determined, and would undoubtably depend on the population one is interested in (e.g., age, problem domain). The present study evaluated three indices of attentional bias that are based on differences scores between or differential probability of responding in congruent vs. incongruent trials. Alternative computational methods, like drift-diffusion modeling, are found to yield improved reliability estimates for a verbal dot probe test in adults with clinical anxiety. The index computed following this approach is considered by the authors to be a more precise measure of attentional bias than the traditional ABS (Price et al., 2019). However, this approach has not been evaluated with a pictorial food-related dot probe test, nor in a sample of youth.

To conclude, the present study could not provide evidence for the use of this particular version of the dot probe test to assess food-related attentional bias in youth with and without obesity. These results warn against the ill-considered and casual use of a dot probe task in experimental or correlational research, and again display the need to carefully scrutinize the psychometric properties of the test in the same meticulous way they would evaluate the psychometric properties of other measures (i.e., questionnaires) (Rodebaugh et al., 2016; De Schryver et al., 2018a; Parsons et al., 2018). If researchers would decide on reporting results of the dot probe task, they are urgently and insistently encouraged to also report, evaluate and discuss the psychometric characteristics (e.g., reliability of indices, correlations between general RT and indices) of their test version for their sample.

## Data Availability Statement

The raw data supporting the conclusions of this article will be made available by the authors, without undue reservation.

## Ethics Statement

The studies involving human participants were reviewed and approved by Ghent University FPPW. Written informed consent to participate in this study was provided by the participants' legal guardian/next of kin.

## Author Contributions

LV and TN share project administration. LV and MD contributed to conception and design of the study. LV and MB did the literature search. TN collected data and wrote down the data management plan. MB organized and prepared the database. LV, MB, and MD performed the statistical analysis and wrote sections of the manuscript. LV wrote the first draft of the manuscript. All authors contributed to manuscript revision, read, and approved the submitted version.

## Conflict of Interest

The authors declare that the research was conducted in the absence of any commercial or financial relationships that could be construed as a potential conflict of interest.
